# Exploratory multi-methods evaluation of an online intervention for carers of people with high-grade glioma

**DOI:** 10.1093/nop/npad032

**Published:** 2023-06-20

**Authors:** Helen M Haydon, Alethea Blackler, Anna K Nowak, Danette Langbecker, Justin Collier, Georgia Halkett

**Affiliations:** Centre for Online Health, The University of Queensland, Princess Alexandra Hospital, Woolloongabba, Queensland, Australia; Centre for Health Services Research, The University of Queensland, Princess Alexandra Hospital, Woolloongabba, Queensland, Australia; School of Design, Queensland University of Technology, Brisbane City, Queensland, Australia; School of Medicine, University of Western Australia, Perth, Western Australia, Australia; Centre for Online Health, The University of Queensland, Princess Alexandra Hospital, Woolloongabba, Queensland, Australia; Centre for Health Services Research, The University of Queensland, Princess Alexandra Hospital, Woolloongabba, Queensland, Australia; Centre for Online Health, The University of Queensland, Princess Alexandra Hospital, Woolloongabba, Queensland, Australia; Centre for Health Services Research, The University of Queensland, Princess Alexandra Hospital, Woolloongabba, Queensland, Australia; School of Nursing, Curtin University, Bently Western Australia, Australia

**Keywords:** brain cancer, carer needs, digital health, neuro-oncology, online intervention

## Abstract

**Background:**

Inadequate knowledge and skills and a lack of confidence to provide care have been identified as major unmet needs for carers of people with brain cancer. An online intervention was developed to address the unmet needs of carers of people with high-grade glioma.

**Methods:**

Ten carers evaluated the intervention through multiple methods. Acceptability and usability were measured through online data analytics (unique page views, time on page), surveys, and interviews. Questionnaires measured potential impacts on distress (Distress Thermometer), depression, anxiety (Hospital Anxiety and Depression Scale), carer competence (Carer Competence Scale), carer preparedness (Caregiving Preparedness Scale), unmet needs (Supportive Care Needs Scale – Brain Tumor Specific for carers), usability and acceptability (USE).

**Results:**

Results suggested the intervention had high levels of usability (usability scales’ means range = 5.1 to 6.7 out of 7) and acceptability (*M* = 76.3/100). Correlations indicated the potential to impact depression. Qualitative findings highlighted benefits of the intervention as a comprehensive reliable resource that could validate and normalize carer experiences. Interview findings guided further improvements (eg, additional carer videos, content organization).

**Conclusions:**

The study indicated high acceptability and usability of an online intervention for carers of people with high-grade glioma. This exploratory study also provided preliminary indications of a potential to decrease depression. However, a more robust, potentially longitudinal, investigation is needed with a larger and broader sample. Informed by this study, the intervention has been amended and a randomized controlled trial will further evaluate the enhanced intervention.

Primary brain tumors incur a significant symptom burden. As a result of the tumor patients may experience physical symptoms such as weakness and motor dysfunction,^1^ seizures, daytime somnolence,^2^ impaired speech, hearing, and headaches.^3^ Cognitive impairment is a common and devastating symptom, affecting approximately 90% of brain tumor patients at diagnosis,^4^ which can result in neurobehavioral symptoms such as disinhibition and lack of understanding of social cues, reduced motivation, reduced empathy, and other personality changes.^5^ Cognitive impairments and neurobehavioral symptoms limit patients’ abilities to work, drive a car, or undertake their usual family or social roles.^6^

These changes place a significant burden upon patients’ families who must take on challenging caregiving responsibilities, often with little prior understanding of how to provide physical care, manage behavioral changes, or adapt to their own and their relative’s changed roles.^7,8^ Treatment frequently involves complex pharmacological regimens, requiring carers to administer and monitor these medications.^9^ Carers may also need to make ongoing and challenging medical decisions about the patients’ treatment and care.^8^ It is perhaps not surprising given these factors, that 40%^10^ to 52%^11^ of carers report anxiety and 72% report elevated stress levels.^12^

Inadequate knowledge and skills and a lack of confidence to provide care have been identified as major unmet needs for many cancer carers, and may intensify distress.^13^ Among carers of brain tumor patients, a consistent need for additional, and more detailed, information has been found across studies.^9,14,15^ Carers frequently highlight being “unprepared” for their new carer role,^16^ and need information about how to manage physical, cognitive and personality changes,^10,17^ provide day-to-day care, and manage side-effects at home.^7^ Carers report needing help with accessing information about the benefits and side-effects of treatments,^10^ dealing with anxiety and stress, and coping with changes in their own social, work, and family life.^18^

A nurse-led tailored home-based education and support program for carers of patients with high-grade glioma (HGG), *Confidence to care,* was developed by a multidisciplinary oncology team led by members of our team author and author *(deidentified for review purposes)* and has shown promise in initial trials.^19,20^ For instance, the randomized controlled trial (RCT) was effective in improving carer preparedness.^21^ To broaden the reach, and in view of a lack of online programs,^22^ the evidence-based Confidence to Care resources were transformed into an online format and adapted to form a stand-alone asynchronous online intervention (a website). The development of an online program was an extension of the more costly and geographically restricted nurse-led program. Although minimal, there is some evidence regarding online resources for adults living with brain cancer.^22^ While there are evidence-based interventions that have an online component, there appears to be little or no research demonstrating the effectiveness of stand-alone online programs for carers of people with brain tumors.^22^ This current stand-alone program is a web-based resource that can be accessed without barriers, at any time or place.

While there appears to be little or no research demonstrating the effectiveness of stand-alone websites for carers of people with brain tumors,^22^ promising results are emerging for online resources for carers of people living with dementia.^23^ Online resources have broader population reach and are less costly to implement.^23^ This pilot is the prelude to more rigorously examining the effectiveness of the online program in meeting carer support needs.

This online program now incorporates a range of other evidence-based resources, and is no longer nurse-led, but an online portal for carers to explore at their convenience. The objective of the online version is to increase access to evidence-based materials which carers can explore at their own pace within the convenience of their home. The aim of this study was to pilot the online intervention, specifically examining: Acceptability, usability, and how it met carer expectations; and any potential impacts on mood (eg, distress, depression), unmet needs, carer preparedness and competence, and the caring experience in general.

## Methods

A single-group pilot study involving pre- and post-intervention questionnaires and an interview post-intervention was conducted.

### The Caring for the Carer intervention

The online program, Caring for the Carer, was developed with the involvement of consumer representatives It is a 6-module, password-protected, online interactive psychoeducational program designed to prepare carers of people with HGG to undertake caregiving responsibilities. The intervention is guided by Bandura’s social cognitive theory, which proposes that a high degree of self-efficacy increases the likelihood that caregiving tasks will be successfully performed,^24^ and the transactional model of coping, which considers the capability of the carer and the resources that they need to adapt to their situation.^25^ The online program focuses on building carers’ knowledge and skills to perform caregiving tasks, to enhance carers’ confidence and capacity to care for their family members and cope with their situation. The intervention content is divided into modules, to enable carers to proceed at their own pace, guided by self-assessment activities that suggest modules that may be of most help to the carer. The 6 modules include: Understanding HGG, Navigating the System, Changes to Expect, What else can I do?, People around you and When Things don’t go well. The modules consist of: Evidence-based information; links to resources, case examples and; an electronic notebook that allows participants to take notes as needed and personalize some of the information and activities.

### Study Participants

Participants (18 years and above) were eligible if caring for an adult diagnosed with a high-grade (grade III or IV) glioma. Patients may have been diagnosed initially with a low-grade glioma (grade II) that progressed or transformed into HGG. Participants needed to be living with the person they were caring for and resident in Australia. Participants were also required to have internet access, an email address, and adequate English to be able to comprehensively review the online intervention. Carers were ineligible if the person with a brain tumor was less than 18 years old.

### Procedures

The study was approved by the University of Queensland Human Research Ethics Committee (number 2018002631) and reciprocal approval was gained from Curtin University (HRE2019-0279). Recruitment of participants took place between September 2020 and February 2021. Online flyers, social media text and posts and, newsletter articles were distributed via various methods to invite participation. Potential participants were informed of the study via social networking sites (eg Twitter, Facebook) and requests to participate were disseminated via cancer and brain tumor support organizations’ email lists and consumer newsletters. Interested carers contacted the research team and underwent a screening telephone interview. Participant information and consent forms were emailed and written informed consent was gained. Consenting participants were emailed a pre-intervention questionnaire link.

After completing the pre-intervention questionnaire, participants were emailed the link to the online intervention and provided a unique username and password. A follow-up phone call checked participants were able to access the online intervention. Google analytics tracked data usage during a 4-week timeframe. Participants were emailed after 2 weeks to check for any questions or concerns. Participants were emailed the post-intervention questionnaire link 4 weeks after login access was provided.

Participants were also invited to participate in a semi-structured telephone interview. A researcher (author JC), under the supervision of author HH, an experienced qualitative researcher and Psychologist, conducted the interviews. All interviews were conducted by phone with people who had used the online program during the 4-week pre- and post-implementation period. The transcriptions were deidentified and names were replaced with pseudonyms. The interview transcripts were entered into NVivo12 (QSR International, Burlington MA, USA; https://www.qsrinternational.com/nvivo-qualitative-data-analysis-software/home) for data management and to facilitate qualitative analysis.

### Measures

#### Pre-intervention Questionnaire

The distress thermometer (DT)^26^ was used as a single item, point-in-time measure of distress. The participant is asked to estimate how distressed they felt in the previous week by choosing a number between 0 and 10, where 0 equals “No distress” and 10 means “Extreme distress.” The DT shows good validity against a range of standardized measures and across time.^26^

The 14-item Hospital Anxiety and Depression Scale (HADS),^27^ assessed anxiety (7 items) and depression (7 items). Scores (ranging between 0 and 42) result in classifications of “possible” or “probable” mental health disturbances with calculation of a global score ≥ 15 indicating “emotional distress.”

An 11-item Supportive Care Needs Scale – Brain Tumor Specific instrument for carers (SCNS-P&C)^10,28^ measured participants’ brain tumor-related unmet needs that had occurred over the previous month. It is measured on a 5-point Likert scale ranging from no need through to high need.

The Carer Competence Scale (CCS),^29^ a 4-item scale, assessed carers’ perceived adequacy in performing caring activities on a 4-point Likert scale (ranging from “not at all” to “very much”).

Carer preparedness prior to viewing the website was measured using the Caregiving Preparedness Scale (CPS),^30^ an 8-item scale that measures carer readiness for the tasks and demands involved in a caregiving role. The CPS has good internal and external reliability and good construct validity.^31^

Carer demographics, tumor type, and date of diagnosis information were also collected.

#### Post-intervention Questionnaire

To compare results pre- and post-intervention, the post-intervention questionnaire readministered the DT, CPS, CCS, HADS, and SCNS-BT. Additional measures post-intervention included: technology self-efficacy; intervention usability; and acceptability.

The 10-item Modified Computer Self-Efficacy Scale^32^ assessed participant technology use competency. This scale has high internal reliability (*α* = 0.94)^32^ and has been administered in an Australian population. Scores range between 10 and 100, and measures self-efficacy in using technology alone and with the support of others.^32^

The USE questionnaire^33^ assessed 4 domains of perceived usability of the online intervention: Usefulness; ease of use; ease of learning and satisfaction. The 30-item scale has been reported to have excellent internal reliability (*α* = 0.98), with good construct validity when compared with other usability scales.^34^

Despite the importance of assessing the acceptability of a health intervention, there remains a lack of definition and validated assessment in this regard.^35^ As such, we developed a questionnaire based on Sekhon et al.’s (2017) theoretical framework of acceptability.^35^ Each item was scored on a 7-point Likert scale (strongly disagree to strongly agree), and scores were summed to create an index, which was transformed to a 0–100 scale for ease of interpretation.

#### Post-intervention Interviews

The semi-structured interview sought to understand the acceptability, usability, and usefulness of the online intervention in greater depth. The phone interviews with individual users of Caring for the Carer ranged from 19 to 61 minutes (*M* = 30 minutes). Interviews were recorded and transcribed verbatim.

#### Google Analytics

Engagement with the intervention (in the form of unique page views and time on each page) was assessed using Google Analytics. These raw data were later coded into thematic groups, as explained on P. 9 and in Table 1.

**Table 1.** Coding Scheme for Analytics Data

Code	Description
Helpful links	Helpful links page
Navigation	Navigation assistance and how to use the website
Registration	Registration administration
Search	Search and search results
Carer well-being	Advice on carer’s caring for themselves
Practical advice	Practical advice on managing caring tasks
End of life	Information and advice on end of life
Health and support systems	Information and advice on accessing and using health and support systems
Info about HGG	Information about high-grade gliomas
Notes	A notes page provided for participants to make their own notes while using the website
Stories	Carer stories used as examples.

### Analysis

#### Survey Data

Where participants answered fewer than half the items on a scale, their scores were omitted. Mean imputation was not needed because the remaining participants answered all items. Due to the small sample size, survey data were analyzed descriptively. Continuous data were summarized using means and medians, depending on normality of the data. Categorical data were summarized using percentages. Bivariate statistics (paired t-test and Wilcoxon rank sum test, depending on variable normality) were used to identify the relationship between variables. Cohen’s d was calculated to provide an approximate effect size for the change over time in variables measured both pre- and post-intervention. Effect sizes of *d* = 0.2, *d* = 0.5, and *d* = 0.8 were interpreted as small, medium, and large effects, respectively.^36^ Statistical significance was assumed at *P* < .05.

#### Interview Data

Two researchers conducted a thematic analysis of the transcribed interviews as guided by Braun and Clark.^37^ Each researcher compared and contrasted codes within and across each interview until first-level themes were drafted. One researcher (author JC, a 30-year-old male who conducted all of the interviews and had qualitative analysis experience) analyzed all interviews and the second researcher (a 28-year-old female with qualitative research experience) analyzed 4 interviews. Once first-level themes were drafted, 3 rounds of peer debriefing were conducted with the researchers and author HH. These discussions lead to the final higher-level themes.

#### Google Analytics

Google analytics data for number of page views and average time on pages were collected for 257 unique URLs contained within the website. These data were coded in MS Excel into 9 categories, as per the coding scheme in Table 1.

## Results

### Survey

#### Survey participants.

Of 16 people who consented, 1 did not commence the study. Of the remaining 15 participants, only 12 people completed the post-intervention survey. Of these 12, one was excluded due to never logging onto the online intervention and another was excluded as the person they cared for went into a residential setting, which meant that the intervention potentially had less relevance in this type of caring. See Supplementary Figure 1, for a diagrammatic representation of survey recruitment.

#### Survey results.

The survey results suggest good acceptability, with at least 8 participants completing all items for all scales at each time point. The median times to complete the pre-intervention and post-intervention surveys were 9 (min 6.3, max 42.5) and 17 (min.6 max 61.6) minutes, respectively. The average time between completion of the baseline and follow-up surveys was 38 (SD 11.9) days.

All scales indicate acceptable reliability (Cronbach’s alpha ≥ 0.70)^38^ at each time point with the exception of the CCS at follow-up, where a negative average covariance between items was observed at follow-up.

Descriptive statistics for the normally distributed (Table 2) and not normally distributed variables (Table 3) are presented. Mean scores on the DT (*M*^diff^ = 1.3, SD = 2.4, *P* = .11), and HADS overall (*M*^diff^ = 3.2, SD = 4.6, *P* = .06) and anxiety (*M*^diff^ = 1.1, SD = 3.2, *P* = .31) and depression scales (*M*^diff^ = 2.1, SD = 2.3, *P* = .019) decreased over time. Although Cohen’s d and statistical significance were calculated, as the sample size was small, results should be interpreted with caution. As such, these results are not included as part of the main manuscript but can be viewed in Supplementary File 2. The mean number of moderate or high unmet brain tumor-specific caregiving needs also decreased over time (*M*^diff^ = 1.2, SD = 0.7, *P* = .13). The mean carer competence score decreased slightly over time (*M*^diff^ = 0.1, SD = 0.3, *P* = .40), indicating a decline in perceived competence to provide care. The median preparedness to care score increased over time, indicating an increase in preparedness to provide care (Mdn^diff^ = 4.7, *p* = 0.48).

**Table 2.** Descriptive Statistics for the Normally Distributed Variables

Variable	Baseline (*N* = 10)	Follow-up (*N* = 10)	Difference	
	Mean (SD)	Mean (SD)	Mean (SD)	*P*-value
Distress thermometer *(0–10 scale, higher scores indicating higher distress)*	6.8 (1.6)	5.5 (2.4)	1.3 (2.4)	.11
HADS Anxiety *(0–21, higher scores indicating greater anxiety symptoms)*	12.6 (3.8)	11.5 (3.2)	1.1 (3.2)	.31
HADS Depression *(0–21, higher scores indicating greater depressive symptoms)*	10.8(5.0)	8.7 (3.9)	2.1 (2.3)	.02
HADS Total *(0–42, higher scores indicating greater symptoms)*	23.4 (7.6)	20.2 (6.0)	3.2 (4.6)	.06
Number of moderate or high-level unmet brain tumor caregiving needs *(0–11 unmet needs)*	6.1 (3.2)	4.9 (3.5)	1.2 (0.7)	.13
Carer competence *(0–4, higher scores indicating greater perceived competence)*	3.3 (0.4)	3.2 (0.3)	0.1 (0.1)	.40

**Table 3.** Descriptive Statistics for Variables Not Normally Distributed

Variable	Baseline (*N* = 10)	Follow-up (*N* = 9)	Change (*N* = 9)	Test statistic	*P value*
	Median (min, max)	Median (min, max)	Median (min, max)		
Preparedness to care *(0–32, higher scores indicating higher preparedness)*	12.3 (1.3, 23.3)	18.7 (1.3, 24.7)	4.7 (-20.0, 13.3)	Z = −0.712	.48

Scores on post-implementation only variables (see Table 4) indicated that participants viewed the online intervention ease of learning most highly (*M* = 6.7, SD = 0.4), with ease of use (*M* = 5.2, SD = 0.8), usefulness (*M* = 5.2, SD = 1.2) and satisfaction scores (*M* = 5.1, SD = 1.0) generating means above 5 on a 7-point scale and indicating a positive rating of the program.^34^ Mean acceptability of the program was 76 (standard deviation 13.01) on a 0–100 scale, also suggesting the program was acceptable to participants.

**Table 4.** Post-implementation Only Variables

Variable	*N*	Mean (SD)
Technology self-efficacy *(10****–****100, higher scores indicate greater technology self-efficacy)*	9	85.2 (14.3)
USE - Online intervention Usefulness *(1****–****7, higher scores indicate greater usefulness)*	9	5.2 (1.2)
USE - Online intervention Ease of Use *(1****–****7, higher scores indicate greater ease)*	10	5.2 (0.8)
USE - Online intervention Ease of learning *(1****–****7, higher scores indicate greater ease)*	9	6.7 (0.4)
USE - Online intervention Satisfaction *(1****–****7, higher scores indicate greater satisfaction)*	9	5.1 (1.0)
Acceptability of the program *(0–100, higher scores indicate greater acceptability)*	9	76.3 (13.0)

### Analytics

**Figure 1.**
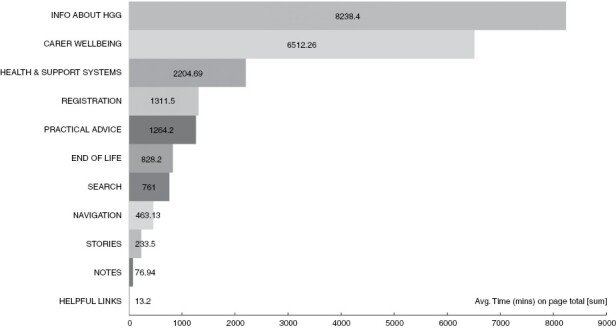
Meantime (seconds) on pages (coded by the eleven themes) over the period of the intervention.

**Figure 2.**
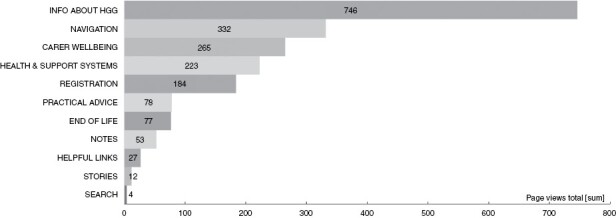
Number of page views (coded by the eleven themes) over the period of the intervention.

Google analytics returned results on 257 separate URL pages. Figure 1 shows the mean time spent on these pages over the intervention of these results. Most time was spent on pages and most views were related to information about HGG (Figure 2). For detail regarding the content of these pages, please see Table 1.

### Interview Findings

#### Interview participants.

Eleven (8 females, 3 males) carers, aged 47 to 63 (*M* = 55) years of age, were interviewed. All except one were spousal carers. Although there was only a small sample size, data saturation^39^ was reached with a steep decline in new codes toward the end of analysis and the peer debriefing meetings came to a consensus that the central themes for this pilot had been generated. The range of time caring for a person with a brain tumor ranged from 6 months to 6.5 years (mean= 2 years and 3 months). Participants were from Queensland (*n* = 4), Australian Capital Territory (*n* = 3), New South Wales (*n* = 2), Victoria (*n* = 1), and Western Australia (*n* = 1).

#### Themes.

The thematic analysis generated 2 overarching themes: One, how the online intervention met carer expectations and 2, perceived impact of the online intervention. The theme regarding how the online intervention met carer expectations was comprised of 3 subthemes. That is, participants perceived that: (1) the intervention was relevant to their needs and comprehensive enough so that all such information could be located in the program, (2) their needs were satisfied overall, but with some desire for more in-depth information addressing the needs of more experienced carers, and (3) some improvements in the visual design was needed. The second theme highlighted perceived impacts of Caring for the Carer and was also comprised of 3 subthemes. That is, participants reported that: (1) the online intervention validated their caring efforts and provided validation that they were doing as much as they could even when they wished they could do more, (2) the intervention helped normalize their situation, especially as a result of being exposed to other carer experiences, and (3) the program prompted reflection upon their own carer journey and experiences.

#### How the online intervention met carer expectations.

##### Relevant and “Everything in one spot”.

All 11 carers described satisfaction with the Caring for the Carer online intervention, stating that the intervention and its content were informative and relevant. Some participants conveyed satisfaction that the program was comprehensive with information “where everything was in one spot” [C6]. The participants reported (see examples below) that the online intervention was relevant to their caring needs and was a primary place to go for information.

“It was extremely informative.” [C16]

“I went through pretty much everything and I think all of it was excellent information.” [C9]

The following quote shows the perception that the content is potentially relevant across carers.

“As a carer there will become a time where I would argue all the carers would want to read it.” [C6].

### Content Tailored to Address the Needs of More Experienced Carers

Although participants voiced satisfaction with the online intervention some, who had been caring for a longer time, expressed a desire for more in-depth content. They described how they found some of the information too basic and probably more suited for people closer to diagnosis. As described above, this study’s cohort had been caring for an average of 2 years and 3 months.

For instance, one participant highlighted how useful and comforting having access to such a program would have been at the beginning of their caring journey but implies that it is less useful in their current situation years later.

“I really felt that, if that had been around, at the beginning of my three years, it would have just saved me an enormous amount of time. In terms of me being obsessed, and confused and terrified and about, you know, the whole disease.” [C10].

More overtly, the following 2 quotes demonstrate the desire for more in-depth information and reiterate the concept that they would have had more use for the information at the beginning of their caring journey.

“I did think they were too shallow. I felt like they didn’t have the depth that I’d be wanting for my personal self. Having said that, if I was right at the beginning again, like twelve months ago. Then I probably would. I think they’d be valuable as a first step to look at but then I think as you start progressing through and you have more and more questions you come looking for more.” [C6].

“Commonly, I found that as I went to each section, I would go, “I already know that”. I said this to you when we first started to go into this trial, because I’m two years into his disease, my perspective of what that website’s telling me is going to very different to someone who’s brand new into it. They might find all of this highly informative, knowledge and power-bringing to themselves, because it’s really putting them in a seat where they’re starting to understand more about the disease. You don’t get that information when you go in for a ten-minute clinical appointment with your oncology team.” [C15].

### Improvement Needed in Visual Design

Several participants highlighted the need to integrate more interactive elements and make the online intervention more visually appealing. Carers were aware that the online intervention was in its infancy and commented that it could be made more appealing through improvements in its visual design. They offered that it was “flat in its appearance” [C1] and needed more visual appeal through animations or more graphics.

“…visual side of it, perhaps, but I know it’s early days so it’s hard to say, because some people more visual than others and …have different categories or something I’m not sure, yeah… like having more graphics, or that kind of thing.” [C9]

“You know if this isn’t the end state product. That you’d say that if there’s severe enhancements, that it’s to evolve would, would be about the visualization, the user interaction, with it is quite, I’d call it flat in its appearance.” [C1]

“I tend to go to look at sites that have more animations or more caring type tones about it… [more visual] But, still with the context of information being within. For example, I’ve got it open now, on the first main page for the website, it almost opens up like a news article to me. Whereas, other pages that I’ve gone to do look at things might have a picture of a family, or a picture of a peaceful scenery type thing, and it’ll talk about going to look for different things. I don’t think it’s warm and inviting, I think it’s actually quite – like, news-like…” [C15]

### Perceived Impact of the Online Intervention

When asked about its utility, participants described the impact of the online intervention on their thoughts and actions. The 3 subthemes generated were: Feelings of validation; normalization of carer experiences through learning about other carer experiences and: Reflection or increased awareness regarding their own situation.

#### Validation

An important impact on carers using the online intervention was validation. That is, while the information may not have provided substantial new insights into their caring experiences (as per the finding that this cohort of carers were more experienced and searching for something more in-depth) it provided validation regarding what they were already doing as more experienced carers. Participants described how, in a situation of uncertainty and distress, the online intervention was reassuring as it often reinforced that they were doing well in their caring efforts. Statements alluding to self-doubt were accompanied by assertions that the online intervention validated that the way in which they were caring was probably the best they could do.

“It was almost comforting. Almost, cause [sic] there is nothing that comforts me in this case.” [C5]

“It allowed me to recalibrate [SIC], to tick the boxes in my own head that I was doing all I could…it was reassuring” [C6].

“I think, it has a positive effect. Because, it has that timeout, to think oh you know okay … I can do this and you know like other people have been here and it’s ok. So that, I feel it’s, what’s the word, it’s a reinforcement of that you are on the right [track]. You are trying to do the right thing by your whoever it is, your loved one.” [C9].

#### Normalization

Through reading about other people’s caring experiences on the intervention, carers described how they felt reassured that they weren’t the only ones going through this caring experience. Participant responses indicated that Caring for the Carer, particularly exposure to other carer stories, could normalize a carer’s situation, experiences, and feelings. The intervention’s focus on caring for someone with a brain tumor could provide reassurance that the carer was not alone in their plight. For instance, C9 stated, “other people have been here and it’s ok.”

“You realise that you’re not the only one who’s going through this. And that there’s support out there.” [C3].

#### Reflection

Carers mentioned how the online intervention “triggered” self-reflection, which they sometimes acted upon. Reading about other people’s experiences leads to reflection on their own situation and normalization. The online intervention, and subsequent reflection, prompted some carers to act. For instance, one carer who had read about the benefits of “laughter” even in the face of a distressing situation was prompted to seek out what made those around her laugh. Another participant relayed how they had shared some useful information with their adult children.

“One of the tips was to find something that made you laugh and I haven’t come across that before. That’s the first time I’ve come across that and that sent me off on trying looking at bloopers and outtakes from movies. [I: Oh good it prompted you.] Yes, because it made me realize that it’s a long time since I had anything to laugh about… And having the tip to just find something that made me laugh. I actually asked my kids, who are grownup adults and I said come on guys, what used to make mum laugh?” [C3].

## Discussion

Emerging research shows that online interventions have the potential to efficiently support carers of someone with dementia.^23^ Yet research is still lacking on the effectiveness of these online interventions,^23^ with little to no research on stand-alone web-based interventions for carers of someone with neuro-oncological disorders. This current research provided a pilot evaluation of an online program for carers of people with a brain tumor. Overall, the piloting of the online intervention, Caring for the Carers indicated that it was acceptable and usable. Combining survey and interview results, the pilot suggested the intervention was useful and had the potential to positively impact carers (eg, on mood, unmet needs, and validation of experiences). However, randomization would be needed to demonstrate whether these changes occur over time or are a result of the intervention. Initial results indicate that the website has the potential to address the need for online information for carers of brain cancer patients as highlighted in Schaefer et al.’s (2021) systematic review.^22^

### Usability

Survey results indicated that the intervention was easy to learn to use, acceptable and useful. The computer self-efficacy^32^ results showed that the sample was fairly confident in using technology, which would also explain the ease of use results. In interviews, the carers described the intervention as relevant to their caring experiences, informative, and useful to have reliable information all in one spot. The importance of usability and acceptability of an online program for carer of people with cancer cannot be understated^40^ as it aims to address the unmet needs of this cohort.^22^ The moderate reduction in unmet needs, even in this small sample, supports further study to confirm the utility of the intervention.

However, participants also expressed a desire for more in-depth information to meet the needs of carers who have been caring for a longer period of time. While noteworthy, such findings may also be an artifact of the current sample of carers being further along on the caring journey (average of 2 ¼ years) and highlights the need for further examination with a broader group of carers. This result and the Schaefer et al. (2021) review show that the usability of the pilot website needs amending to allow ease of navigation as a function of the cancer care continuum. This current study did, however, indicate that the content validated caring experiences for those further along the caring trajectory.

The interviews also provided in-depth feedback regarding possible improvements to the intervention. For example, some carers suggested improvements to the layout and visual design, which have been incorporated into the next iteration of the intervention.

### Potential Impact on Mood and Confidence

The quantitative results indicated a reduction in depression and a moderate reduction in distress and anxiety following the intervention, as well as an increase in perceived carer preparedness. However, considering the small sample size, such results are to be viewed with caution. An RCT would help to determine whether the intervention was the main contributor to these changes or whether change over time would have occurred without the intervention. Heinsch et al.’s (2021) systematic review found that significant clinical impact of similar interventions are a rarity and also calls for more RCTs.^41^

The interview findings indicated the presence of self-doubt. Carer self-doubt pertained to fears that were not doing enough to care for the person with a brain tumor. Confidence and preparedness in carers can influence communication with health professionals and patient wellbeing,^42^ yet helplessness can be a common issue especially when faced with palliative conditions.^43,44^ The interview findings suggested that the self-doubt was countered by feelings of validation as a result of engaging with the intervention. Further examination of the role of self-doubt among carers is needed as well as the relationship between general self-confidence and confidence to care.

The interview findings underlined how the intervention could assist in validating the emotions associated with caring, and normalizing their situation. The importance of validation of one’s subjective experience and emotions has long been established^45^ as it can have a marked effect on a person’s emotional response to a situation.^46^ Validation acknowledges the reality and potential distress of an issue such as caring for someone with cancer, and provides “permission” to feel; however, they feel.^47,48^ According to Linehan’s (1993, 2015) conceptualization of validation, normalization is a component that allows a person to feel that their experience and emotions are “normal” and likely to be experienced by others in similar situations. The videos and case studies in the online intervention showed carers that there were others who had similar reactions to caring for someone with a brain tumor, thus validating and normalizing their experience.

### Reflection^1^

Interview findings also suggested that the online intervention prompted self-reflection or reflection on their situation, which, in turn, often triggered a behavioral response. Reflection is a metacognitive process that involves unbiased self-observation of one’s own attitudes, thoughts, and behaviors to gain insight and increase personal effectiveness.^49^ It can involve an attempt to understand one’s reaction to an event and make sense of the resulting feelings.^50^ Research shows that adaptive reflection can assist with coping in a distressful situation, reducing negative emotions and increasing well-being.^50^ Consistent with the current study, existing research also demonstrates how reflection can promote well-being and prompt behavior changes in caregivers^51^ associated with an improved carer experience.^52^ Furthermore, as found in the current study, digital interventions have the potential to prompt and “guide” self-reflection so that it is adaptive and supports carers to “make sense” of their situation and potentially lead to adaptive coping behaviors.^51^

### Traffic on the Intervention Pages

The analytics data were coded for greater coherence and clarity. Google analytics showed that seeking information about HGG, carer well-being, and health and support systems were the most common activities. This supports the need for information that is easy to access, clear, and useful around these issues. The relatively high numbers for both time on page and page views related to website administration such as registration, navigation, and search are disappointing. We need to make the online intervention easily accessible and the registration and set-up process were too time-consuming. These processes, needed for data tracking, have been reviewed and simplified in the current version and will be completely removed after the final evaluation component project. We have also removed the notes feature, which necessitated much of the security and registration process in order to keep people’s notes private. Notes were difficult to include on many platforms, could have perceived privacy issues, was not well used and there are many other ways in which people may prefer to take and keep notes. Scores for stories about carers were also quite low. However, sections of these stories were also accessible throughout the intervention, where relevant, possibly superseding the need to review the whole story. Some interview participants also said they would prefer real people in the stories. Subsequently, we have created a series of videos of “real” carers speaking about a range of relevant topics and added these to the next iteration of the website.

### Limitations

The main limitation of this pilot study was the small sample size. Sampling methods, focused on recruitment from cancer support groups, likely favored potential participants with higher levels of health literacy and those already seeking resources. However, triangulation of multiple methods makes the study more robust. Unfortunately, one item (“It makes the things I want to accomplish easier to get done”) from the usefulness domain of the 30-item USE questionnaire was not transposed to the online survey and therefore, never asked. The effect on scale reliability is probably minor as there were 7 remaining items measuring usefulness. Finally, although all carers appeared to have insight and provided detail with regard to their caring and the impact of the online intervention, it could not be verified that they were actually carers of someone with HGG. Furthermore, to reduce burden on the carers, we did not collect detailed data (eg, performance status) on the person with HGG.

## Conclusion

The study indicated good acceptability and usability of an online intervention for carers of people with HGGs. A decrease in depression was found between pre- and post-implementation results. As such, an RCT would help to determine whether the intervention was the main contributor to decreased depression. This “one stop shop” was seen as useful and prompted carers to reflect on their own coping and caregiving. However, the carers who evaluated the online intervention provided feedback that they required more in-depth information especially as they were further along the trajectory of care. This highlighted the need for information to be tailored as a function of the care continuum. The online intervention has been amended in response to this study’s findings (eg, additional videos of carer experiences, increased user-friendly design and formatting, arrangement of topics aligning with the caring trajectory from diagnosis) and a follow-up RCT will further evaluate the enhanced intervention.

## Supplementary material

Supplementary material is available online at *Neuro-Oncology* (http://neuro-oncology.oxfordjournals.org/).

npad032_suppl_Supplementary_Material

## Footnotes

1 Reflection and self-reflection are often used interchangeably. (https://support.mips.com.au/home/ahpra-self-reflection-is-good-healthcare-practice).
