# Supplementary material for: Exploratory multi-methods evaluation of an online intervention for carers of people with high-grade glioma
Source: Neurooncol Pract. 2023 Jun 20;10(6):544–54. doi: 10.1093/nop/npad032 (PMC10666811; doi:10.1093/nop/npad032)
Supplement: npad032_suppl_Supplementary_Material [file npad032_suppl_supplementary_material.docx]

# Supplementary File 1


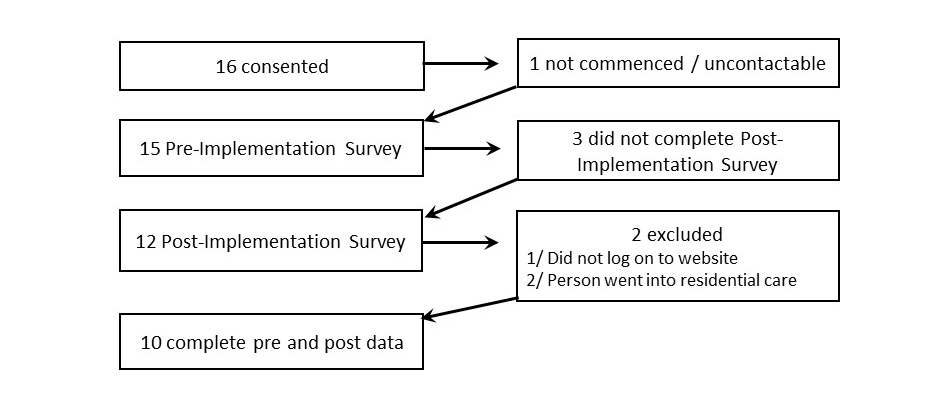


Figure 1. Pre- and post-implementation survey sampling

# Supplementary File 2 – Results including Effect Size and Statistical Significances Reporting

Mean scores on the DT (M^diff^ = 1.3, SD = 2.4, *p* = 0.11), and HADS overall (M^diff^ = 3.2, SD = 4.6, *p* = 0.06) and anxiety (M^diff^ = 1.1, SD = 3.2, *p* = 0.31) and depression scales (M^diff^ = 2.1, SD = 2.3, *p* = 0.019) decreased over time. The size of the effect (Cohen’s d) was large for depression (0.90), moderate for distress (0.55) and small for anxiety (0.34), although only the change in the HADS depression scale demonstrated statistical significance. The mean number of moderate or high unmet brain tumour specific caregiving needs also decreased over time (M^diff^ = 1.2, SD = 0.7, *p* = 0.13), showing a moderate effect size (0.52). The mean carer competence score decreased slightly over time (M^diff^ = 0.1, SD = 0.3, *p* = 0.40), indicating a decline in perceived competence to provide care, although this effect was low (0.28). The median preparedness to care score increased over time, indicating an increase in preparedness to provide care(Mdn^diff^ = 4.7, *p* = 0.48)although this did not reach statistical significance.
